# The Potential of Edible Insects as a Safe, Palatable, and Sustainable Food Source in the European Union

**DOI:** 10.3390/foods13030387

**Published:** 2024-01-24

**Authors:** Ann Conway, Swarna Jaiswal, Amit K. Jaiswal

**Affiliations:** 1 School of Food Science and Environmental Health, Faculty of Sciences and Health, Technological University Dublin—City Campus, Grangegorman, Dublin 7, D07 ADY7, Ireland; c15702861@mytudublin.ie (A.C.); swarna.jaiswal@tudublin.ie (S.J.); 2 Environmental Sustainability and Health Institute, Technological University Dublin—City Campus, Grangegorman, Dublin 7, D07 H6K8, Ireland

**Keywords:** entomophagy, consumer perceptions, consumer concerns, safe food, food hazards, nutritional composition, insect farming, legislation, sustainability

## Abstract

Entomophagy describes the practice of eating insects. Insects are considered extremely nutritious in many countries worldwide. However, there is a lethargic uptake of this practice in Europe where consuming insects and insect-based foodstuffs is often regarded with disgust. Such perceptions and concerns are often due to a lack of exposure to and availability of food-grade insects as a food source and are often driven by neophobia and cultural norms. In recent years, due to accelerating climate change, an urgency to develop alternate safe and sustainable food-sources has emerged. There are currently over 2000 species of insects approved by the World Health Organization as safe to eat and suitable for human consumption. This review article provides an updated overview of the potential of edible insects as a safe, palatable, and sustainable food source. Furthermore, legislation, food safety issues, and the nutritional composition of invertebrates including, but not limited, to crickets (Orthoptera) and mealworms (Coleoptera) are also explored within this review. This article also discusses insect farming methods and the potential upscaling of the industry with regard to future prospects for insects as a sustainable food source. Finally, the topics addressed in this article are areas of potential concern to current and future consumers of edible insects.

## 1. Introduction

Entomophagy, the practice of eating insects, has been a part of human diets from prehistoric times to the present day [1,2,3]. The eggs, larvae, pupae, and adults of certain insects are not only rich in fats, essential fatty acids; protein; essential amino acids; carbohydrates, including chitin and vitamins; and minerals [4], but also potentially present a sustainable alternative to traditional livestock production [5]. Insect farming requires less land, water, and feed, and produces fewer greenhouse gases, thus offering a potential solution to the environmental challenges posed by conventional animal agriculture [6]. Despite these benefits, entomophagy has only recently gained interest in Western countries [5]. In many cultures, insects are often perceived as dirty, disgusting, or as disease vectors, contributing to a neophobic cycle regarding entomophagy [6,7]. However, with the world population predicted to reach nine billion by 2050 and the demand for safe and sustainable food expected to increase by approximately 60% [8,9,10], alternative sustainable protein sources like insects are becoming increasingly important.

In some parts of the world, particularly in Africa, Asia, and Latin America, entomophagy is already a common practice [3]. In contrast, in the European Union, the potential of edible insects as a food source is still being explored, with legislative requirements developed to ensure the safety of all foodstuffs placed on the market [11,12].

**Figure 1:**
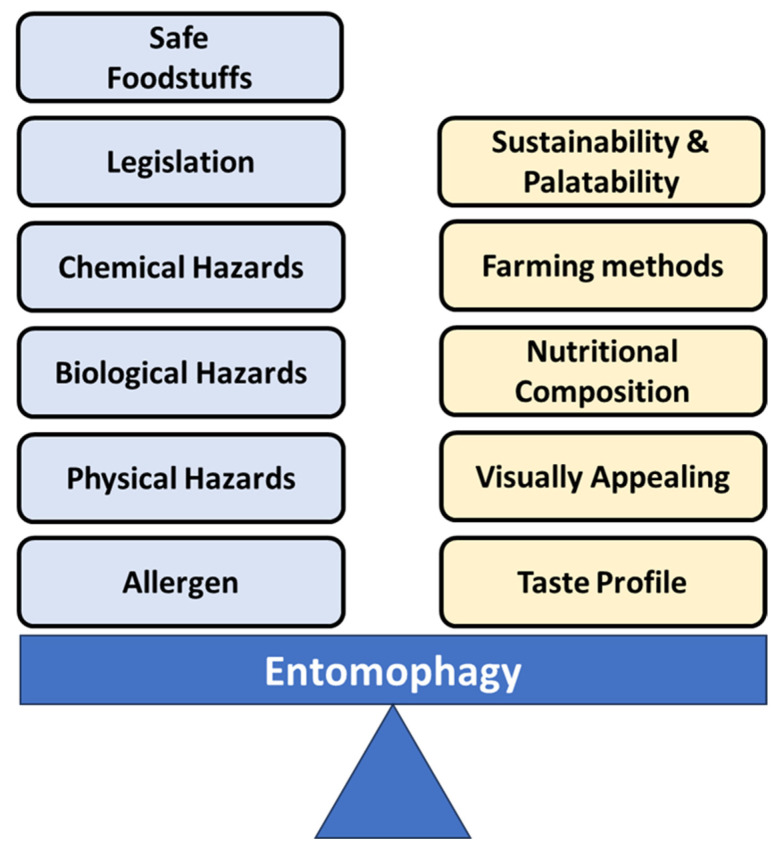
Factors that could potentially affect the safety, palatability, and sustainability of edible insects and insect-based foodstuffs.

Despite the growing body of research on entomophagy, there are still gaps in our understanding of its potential impacts and benefits (Figure 1). Moreover, it must be noted that current research regarding entomophagy may not have been possible without the significant contribution by Meyer-Rochow [4] who identified the potential of edible insects and also effectively initiated further research within this area by suggesting the WHO and FAO support insects as a future food and feed source. Furthermore, limited data is available regarding anti-nutrients which by definition have the potential to hinder or inhibit the absorption of nutrients such as minerals or alternatively to provide antioxidants including but not limited to polyphenols including tannins [13]. Anti-nutrient content within insects can vary between species, where crickets can potentially contain 3159.0 mg/100 g of phytate and 900 mg/100 g of tannin estimated on a dry weight basis, while grasshoppers can potentially contain 1100.1 mg/100 g of phytate and 1050.0 mg/100 g of tannin, also estimated on the basis of dry weight [13]. Furthermore, the anti-nutrient and nutrient content of insects can vary considerably depending on the species under consideration, the species development stage, gender, diet, and application of processing procedures. This current article aims to address these gaps by exploring the nutritional, environmental, and cultural aspects of entomophagy, as well as the food safety concerns related to the consumption of insects, including allergens, gluten, chemical hazards, and microbiological hazards [10,12,13,14,15,16]. Our objective is to provide a comprehensive review of the current state of entomophagy research and to identify potential areas for future study. We hope that our findings will contribute to the ongoing dialogue regarding the role of insects in our food system and help pave the way for the integration of edible insects into a viable and sustainable European foodscape.

## 2. Edible Species

Insects offer a multitude of benefits that make them an attractive option for food consumption [17]. From a health perspective, insects are a rich source of protein, essential amino acids, fats, vitamins, and minerals, making them a highly nutritious food source [18]. Their nutritional profile can contribute to a balanced diet and help combat malnutrition and overnutrition, addressing key health concerns in many parts of the world [19]. Moreover, a recent review by Zhou et al. [20] found there was an annual increase in type 2 diabetes cases worldwide, while Lee et al. [21] agreed that obesity contributed to the increased development of diabetes. Furthermore, obesity increases the risk of developing a variety of diseases, including type 2 diabetes mellitus (T2D), cardiovascular disease, and cancers, all of which can negatively impact quality of life [22]. However, recent research regarding diet-induced obese mice found that Yellow mealworm (*Tenebrio molitor*) and Lesser mealworm (*Alphitobius diapernius*) proteins hindered weight gain and improved the metabolism of the obese mice [23]. Moreover, mealworms and their extracts are considered beneficial for metabolic health and improved metabolism, which may be due to a combination of components therein, including but not limited to protein [23], chitin [24], and fatty acids [25]. In addition, Seo et al. [26] found that Yellow mealworm (*Tenebrio molitor*) larvae extracts also improved metabolism by reducing hepatic steatosis and lowering plasma AST and ALT concentrations. In terms of environmental impact, insect farming presents a more sustainable alternative to traditional livestock farming. It requires less land, water, and feed, and produces fewer greenhouse gases, aligning with global efforts to mitigate climate change and promote sustainable food production. Moreover, certain insect species have shown resistance to various conditions, which could be beneficial in the context of insect farming [27].

Beyond health and environmental considerations, the cultivation of insects also holds socioeconomic benefits. Insect farming can provide income opportunities, particularly in rural areas where economic resources may be limited. This can contribute to poverty reduction and economic development, fostering resilience in vulnerable communities. In fact, a study focusing on the consumption of edible insects in Kinshasa, Congo, in a context of food crisis and shortage, found that individual and collective factors, as well as the context of consumption and emotional factors, influence insect consumption [28]. Thus, the practice of entomophagy, or insect consumption, holds promise not only as a solution to nutritional needs but also as a strategy for sustainable development and economic growth.

Moreover, edible insects can play a significant role in achieving the United Nations Sustainable Development Goals (SDGs), particularly those related to ending poverty, ensuring food security, and promoting health and well-being [29]. Regarding Goal 1, which aims to end poverty in all forms everywhere, insect farming can provide new income opportunities, particularly in rural and impoverished areas, thereby contributing to poverty reduction. In terms of Goal 2, which seeks to end hunger, achieve food security and improved nutrition, and promote sustainable agriculture, insects emerge as a highly nutritious food source that can help combat malnutrition. Their farming requires fewer resources than traditional livestock farming, making it a more sustainable option for food production. This aligns with the goal’s emphasis on sustainable agriculture. Lastly, for Goal 3, which aims to ensure healthy lives and promote well-being for all ages, the nutritional benefits of insects can contribute to overall health and well-being. Their high protein, essential amino acid, fats, vitamins, and mineral content can contribute to a balanced diet and improved health outcomes. Thus, the practice of entomophagy, or insect consumption, holds promise not only as a solution to nutritional needs but also as a strategy for sustainable development and economic growth [30].

Previous peer reviewed research undertaken by a selection of academic authors including Rumpold and Schluter [31], identified a compilation of edible insect species, all of which could potentially be reared within the EU for human consumption (Table 1). Some of these species are currently approved as food-grade within the European Union, including *Tenebrio molitor*, *Alphitobius diaperinus* larvae, *Acheta domesticus*, *Locusta migratoria migratorioides* [32], and *Gryllodes sigillatus* which is currently under consideration. Further research is required to expand a larger selection of edible insects [33,34,35,36,37,38,39,40,41,42,43,44,45,46,47,48,49,50,51,52,53,54,55,56,57,58,59,60,61,62,63,64,65,66,67,68,69,70] into our European cuisine.

Currently under EU Novel Food legislation [32], edible insect species approved for human consumption (Table 2) include mealworms, locusts, and crickets, which are available in their raw state or processed into a foodstuff familiar to the consumer [71,72,73,74]. Patents for other food-grade species within the EU are currently pending with *Gryllodes sigillatus* currently included within this category.

## 3. Palatable Foodstuffs Containing Insects

Recently, Hlongwane [75] reviewed the nutritional composition of edible insects which are considered a palatable and rich source of protein in the traditional cuisine of Africa. Meanwhile, Truck [76] considered the potential safety of frozen and dried insect formulations and Van Itterbeeck [77] posed the question “How Many Edible Insect Species are there”? Furthermore, Linn [78] explored insect-based recipes as a palatable contribution towards a sustainable planet. Moreover, Shelomi [79] explored the potential of edible insects as a future food source by identifying and comparing numerous issues regarding positive and negative engagement with entomophagy. However, it must be noted that edible insects are a safe palatable food source with numerous techno-functional and physiological properties which can potentially enhance the end-produce formulation of edible insects into palatable, familiar and acceptable formulations [80,81]. Moreover, Ververais [82] highlighted the scientific requirements and potential challenges of the risk assessment process involved with the development of novel foods. Furthermore, Schiel [83] reviewed the current legal framework regarding the marketing of food-grade insects within the European Union, while Kohler [84] identified the protein, amino aciid and mineral composition of edible insects from Thailand.

Moreover, edible insects have previously been incorporated into palatable, acceptable, familiar, readily available, processed foodstuffs for consumers in Europe [85]. This project was undertaken with a view to introducing consumers to high quality insect products that offer sustainability via a low carbon footprint when compared with traditional sources of meat products [1,86]. Therefore, in the Netherlands in 2015, early adopters who were senior employees of Jumbo’s, a Dutch national food chain which consisted of 550 branches, decided to stock a selection of insect-based convenience foods (Table 3).

Jumbo’s prevailing interests as forward-thinking retailers translated without difficulty into alignment with the project of insects as food [85]. More recently, the company Ynsect [87] has emerged as a major competitor in the insect and insect-based foodstuffs market with the EU, North America, and Mexico. Ynsect is currently the world leader in natural insect protein and fertilizer production with facilities in France andNorth America. Furthermore, Ynsect have applied for several patents within the EU related to their research. Ynsect have also raised approximately $435 million from leading global investors and are exporting their highly digestible premium protein products worldwide. However, there is currently a paucity of knowledge regarding start-up insect farms, costs, and the duration of expected trading.

## 4. Consumer Perception

There are two distinct psychological reactions to insects as a food source for human consumption. Firstly, in countries where entomophagy is the norm, insects are heralded as a valued protein source and consumed without hesitation; whereas, in non-practicing countries such behaviour can evoke negative reactions aligned with disgust as insects are often perceived as dirty and dangerous [88]. Moreover, in a recent study by Ghosh [17], it was observed that a culturally conditioned acceptability and preference for consuming edible insect species was potentially influenced by cultural norms and further influenced by localized customary taboos. Furthermore, it was also found that the consumers’ inherited perceived and experienced expected sensory characteristics of the taste, odour, visual appearance, and texture of edible insects were varied dependant on the individuals’ initial exposure to entomophagy. Therefore, an historic engagement with entomophagy, either positive or negative, could potentially influence a consumers’ selection, preference, acceptance, and willingness to consume edible insect species in the future.

Consumers are more likely to consume insects and insect-based foodstuffs when they are presented in a convenient and familiar form for example burgers, cookies, or flour [7,78]. These familiar formats help reduce the “ick” factor associated with disgust and encourage the consumer to focus on the nutritional benefits and palatable acceptance of the product [7,19]. Moreover, recent innovation within the sector has produced a selection of palatable insect-based food products which can be viewed in Table 4, Table 5 and Table 6 below.

### 4.1. Neophobia

Food neophobia is described as the avoidance of new or unfamiliar foods. Neophobic tendencies are often an individual choice but can also manifest as a result of cultural influences, a lack of exposure to a particular foodstuff, or inherited beliefs [89]. Previous research has confirmed that consumer familiarity with a product was an important factor for developing a positive attitude regarding the consumption of edible insects [90].

However, European cultures often perceive insects as dirty, disgusting, dangerous pests, or vectors of disease [1,2]. Such negative attitudes could possibly contribute to food neophobia, which, in turn, can negatively influence the consumers perception and willingness to consume insects, even if the food product is visually appetizing and a healthy and sustainable food choice [7,8,30].

The Food Neophobia scale was originally developed by Pliner and Hobden [91], to specifically explore neophobia/neophilia in humans. The Food Neophobia Scale has been used more recently to determine the effect of such phobias associated with the introduction of insects as a possible food source [92].

### 4.2. Cultural Influence on the Consumption of Insects as a Food Source

Currently, there is a lethargic uptake of entomophagy in Europe which is due in part to the lack of insect consumption within the boundaries of cultural norms [51]. Cultural norms can potentially influence everyday behaviours including but not limited to food choices, dress code, religious preferences, and educational aspirations. Moreover, entomophagy is not considered acceptable by many citizens in Europe. Several countries outside Europe, such as those in Africa, Asia, and South America, practice entomophagy on a regular basis and indeed consider several species as a delicacy. Citizens within such environments will naturally expect to consume insects at the table or at a minimum on special occasions. However, Laos and Thailand have experienced a decline in traditional entomophagy practices wherenew forms of disgust and other changes in local food culture were not purely determined by traditional cultural factors. This trend was identified by Muller [93] as a local form of “modernity” and not that of Western-dominated globalization or McDonaldisation [94]. Interestingly, Muller [93] observed that, during the 1980′s and 1990′s, Thailand underwent an economic and industrial boom period and its citizens migrated to the cities where they popularized their food culture through economically driven food processes, which, in turn, facilitated the normalization of entomophagy while simultaneously enhancing capitalism. Therefore, in a current reversal of cultural consumption patterns, rural citizens are reluctant to consume insects while the city dwellers and tourists are content to snack on insects as an accompaniment to beer and other alcoholic drinks [93].

However, historically, entomophagy was eliminated from Europe and it is no longer part of our accepted culinary repertoire. Recent studies have focused on reintroducing insects and insect-based foodstuffs to European cuisine [6,95,96]. Furthermore, as observed by House [86], commercial activity within this sector is prominent in Europe and the Dutch Government are currently encouraging a resurgence in entomophagy in Europe. Furthermore, insects are suitable for consumption and processing into familiar formats including but not limited to “Whole and Roasted”.

## 5. A Safe Food Source

Consumers have numerous concerns related to the safety and sustainability of all food sources available in the marketplace [59,96]. Such concerns have increased in response to accelerated climate change and potential food insecurity due to the predicted rise in the global population by 2050, and, therefore, the safety and sustainability of future food production has become paramount to our survival [30]. Furthermore, hazards associated with everyday foodstuffs can also potentially be present in whole insects and insect-based foodstuffs. However, it must also be noted that there is, simultaneously, an extreme overproduction and overconsumption of foodstuffs, which is a major contributor to the current obesity epidemic [97] and related non-communicable diseases [98]. Meanwhile, another recent survey in Europe indicated that the population of the continent is projected to decline in two-thirds of EU regions by 2050 [99]. Similar demographic trends were also identified in Japan, Taiwan, South Korea, China, and Thailand, where births are declining year on year and deaths are increasing due to aging populations. Therefore, it is also expected that population numbers in Asia will also decrease by 2050 [100]. Such trends could potentially impact current consumption patterns and increase the obesity epidemic.

Insects farmed in Europe, in compliance with current mandatory legislation and food safety standards, as applied to foodstuffs suitable for human consumption, are considered safe to consume [80,81,82]. However, insects as a foodstuff must be constantly monitored for the presence of hazards and contaminants such as heavy metals, veterinary drug residues, pesticide residues, chlorinated pesticides, dioxins, pathogenic bacteria, viruses, parasites, mycotoxins, and allergens as a mechanism to keep our food safe [2,10,82]. 

Food-grade insects should only be sourced, reared, and processed under strict HACCP procedures and subjected to all foodstuff regulations and standards as are mandatory under current legislation within the European Union [32,80,82,101]. Moreover, the potential uptake of any contaminants by insects will ultimately depend on the individual species and the developmental stage of an individual’s life cycle [11].

### 5.1. Chemical Hazards

Chemical hazards found within insects are mostly dependent on habitat and feed contamination, both of which can be controlled through selected farming techniques and dietary control [11]. The contaminants may be present in the substrate used, or within the immediate environment, including but not limited to heavy metals, veterinary drug residues, organohalogen compounds, and pesticide residues [16].

#### 5.1.1. Mycotoxins

Mycotoxins are contaminants of note due to their negative impact on public health and food security. They are secondary metabolites which are produced by phytopathogenic and food spoilage moulds such as *Fusarium*, *Aspergillus*, and *Penicillium* genera [11,102], and are often found in the feed substrate upon which the insects are reared [103]. According to Van Huis [1], the mycotoxins of *Fusarium*, *Aspergillus*, and *Penicillium* can also be present within the gut of an insect, thus indicating the potential of related food safety issues because these toxins could potentially impose acute and chronic effects on humans and animals alike. Furthermore, mycotoxins have also been found in varying concentrations in edible insects [104]. However, mycotoxins are more likely to be found in insect frass due to natural excretion processes [11].

#### 5.1.2. Heavy Metals

Heavy metals accumulation limits are currently specified within Regulation (EC) No. 1881/2006 [105]. The concentration of heavy metals accumulated within insect hosts is dependent on the metal under consideration, the concentration of that particular metal within the substrate, the individual insect species, and the growth stage of the insect in question, also including packaging material used [30,90]. Previously, Camenzuli [104] found that non-essential heavy metals such as cadmium (Cd), lead (Pb), mercury (Hg), and arsenic (As) maintained a positive correlation between the substrate concentration and the insect internal concentration, thus indicating that non-essential heavy metals could possibly accumulate within the insect.

Further research by van der Fels-Klerx [16,106] indicated that cadmium could potentially bioaccumulate to a toxic level in *T. molitor* if the species were fed with a cadmium contaminated substrate. However, the concentration of cadmium was found to decrease during moulting and metamorphosis, which resulted in lower adult bioaccumulation concentrations [107]. Furthermore, Truzzi [108] found that yellow mealworm larvae (*T. molitor*) accumulation of heavy metals was within limits permitted under European Union legislation, while Meyer [11] indicated that further investigations should be undertaken regarding *A. diaperinus* and *A. domesticus* to clarify their potential to bioaccumulate heavy metals as per Regulation (EC) No. 1881/2006. Interestingly no evidence of tin (Sn) accumulations within the insects is available in the published literature currently available.

#### 5.1.3. Veterinary Drugs and Hormones

Lincomycin fermentation residues (LFR) are byproducts associated with the pharmaceutical industry which contain high concentrations of antibiotics. However, Luo [109] found that black soldier fly larvae (BSFL) *H. illucens* could effectively degrade such residues due to the structure of their gut microbiota.

Previously, limited information was available with regards to the presence or accumulation of residual veterinary drugs and hormones within farmed and wild insects [16]. A study undertaken on fly larvae by Lalander [110] focused on the antibiotics roxithromycin and trimethoprim and the antiepileptic drug carbamazepine: no bioaccumulation of these pharmaceuticals was noted. However, in a similar study in the United Kingdom, nicarbazin was detected [111].

Veterinary drugs are often combined with the substrate upon which insects are reared in an effort to effectively distribute antibacterial and antifungal agents to the livestock. This method of distribution reduces insect mortality and infections from pathogens but can ultimately adversely affect the growth and survival of the insects as the antibacterial and antifungal agents can be toxic to the mini-livestock [112].

#### 5.1.4. Pesticide Residues

The current trend of regulated vertical farming, which includes controlled feeding regimes, has the potential to produce pesticide residue-free edible insects [7], although pesticides used in agriculture could possibly be present in the plant material and agricultural wastes that are suitable for use as a substrate during insect production [16].

Previously, a study on chiral fungicides by Lv [113], found that *T. molitor* larvae could metabolize and degrade the fungicide epoxiconazole (log K_ow_ 3.58), while Yin [114] identified similar results in metalaxyl with log K_ow_ 1.65. Furthermore, Houbraken [115] found that the combined results of similar experiments on *T. molitor* also revealed a consistent K_ow_ pattern with no bioaccumulation of any of the pesticides within the larvae.

#### 5.1.5. Accumulation of Pesticides

The accumulation of chlorinated and organophosphorus pesticides in wild harvested insects were highlighted in a recent review by Imathiu [7]. The results indicated that 49.2 µg/kg of chlorinated pesticide and 740.2 µg/kg of organophosphorus pesticides were present in wild locusts caught and sold at a local market in Kuwait. The concentrated accumulations within the insects were due to the presence of pesticides on recently sprayed crops in the insect habitat. Furthermore, the accumulation of chlorinated insecticides, chloropyrifos, chloropyrifos-methyl, and pirimiphos-methyl on corn, which was spiked with the individual pesticides at 2.5 mg/kg on a wet basis, was explored by Purschke [116], with respect to *H. illucens* larvae. It was found that chloropyrifos and chloropyrifos-methyl, at spiked level, accumulated below the EC permitted maximum residue level (MRL) for maize/corn of 3 mg/kg as defined originally in Regulation (EU) No. 396/2005 [117]; however, pirimiphos-methyl in maize/corn accumulated above the 0.5 mg/kg accumulation allowance. It was simultaneously noted that, at the spiked levels, the generation of larval biomass was not affected compared to the experimental control and no relevant absorption or accumulation of insecticides within the larval tissue were observed [116]. However, potential accumulations can be controlled via regulated vertical farming methods [7].

#### 5.1.6. Dioxins

Dioxins and furans (PCDD/F) and dioxin-like polychlorinated biphenyls (dI-PCB’s) are chemicals which were banned under the Stockholm convention [11,118]. However, residues of these chemicals are often found in the environment, soils, and in sediment which could potentially contaminate crops used in the food chain [119]. Research undertaken by Charlton [111] detected PCDD/Fs and dI-PCBs in all the fly larvae analysed, although concentrations were below the EC maximum limit for dioxins allowed in feed materials of animal origin as stated in Regulation (EU) No. 277/2012 [120]. Further investigations by Van der Fels-Klerx [106] with regards to polycyclic aromatic hydrocarbons (PAHs), including benzo[a]pyrene, benz[a]anthracene, benzo[b]fluoranthene, and chrysene, revealed concentrations below 1 to 35 μg/kg as defined in Regulation (EU) No. 835/2001 [121]. However, there are currently no maximum limits for PAHs in animal feed. Further research is required to update dioxin accumulation within insects for food as the compounds are potentially carcinogenic and can damage DNA [11].

### 5.2. Biological Hazards

Microbiological hazards are a major food safety concern in Europe with regards to the consumption of edible insects [12]. The extent of contamination by pathogenic microorganisms depends on many contributing factors such as the insect species, whether it was reared in the wild, in a domestic setting, in a closed farming scenario, or how it was processed, the extent to which it was processed, and the conditions of handling, preparation, and hygiene involved [14,31].

#### 5.2.1. Bacteria

Insects are recognized vectors of pathogenic bacteria which present a potential risk to themselves, insect farmers and at times to edible insect consumers, even in instances where there is no contamination from any identifiable sources [1,31]. Subsequent investigations by Grabowski and Klein [54,73] identified the presence of *Bacillus cereus*, *Staphylococcus aureus*, *Escherichia coli*, *Rickettsiella* sp., *Salmonella*, *Shigella*, and *Campylobacter* in a variety of insects at different life-cycle stages. For example, the darkling beetle *A. diaperinus* is a recognized vector of *Salmonella* and *Escherichia coli* within the poultry industry [33]. *T. molitor* are the larvae of *A. diapernius* and can emerge with inherited pathogenic bacteria if the parent was previously infected [73]. Furthermore, *T. molitor* produced in France and Belgium recently displayed genes with resistance to vancomycin, while larvae produced in the Netherlands, Belgium, and Thailand were found to have a high resistance to aminoglycosides [122].

Furthermore, insects are not usually eviscerated before slaughter, therefore careful attention to HACCP-based processing procedures is required to mitigate any residual opportunistic pathogens that may possibly be present within the gut or on the cuticles of the larvae [123,124]. However, the microbial hazards found within processed edible insects are at substantially lower levels and within European limit specifications than those found in unprocessed edible insects [12]. Moreover, current industrial processing technologies contribute positively and effectively to the overall safety, quality, and sustainability of insect-based food products [2].

#### 5.2.2. Viruses

Arboviruses, which are the cause of human diseases such as dengue, West Nile disease, rift valley fever, hemorrhagic fever, and chikungunya, all possess the ability to replicate within their arthropod vectors [125]. However, the vector competence of insects within food and feed systems has not previously been established [50]. Further research by Eilenberg indicated that a selection of viruses, including norovirus, rotavirus, hepatitis E, and hepatitis A, could possibly be introduced to insects via the substrate within production units allowing transfer beyond the primary production stage. Such findings indicate the necessity for strict HACCP implementation within rearing and processing facilities. Financial burdens resulting from the viral infection of livestock are currently borne by the insect farmer as most viruses associated with the insects are usually only pathogenic to the insect [38]. Such viruses can cause a serious threat to the mini-livestock [126].

#### 5.2.3. Prions

Insects are currently unable to produce prions [2]. However, studies undertaken by Post [127] and later by Lupi [128,129] found that insects were capable of acting as vectors for prions if they were reared on contaminated substrate. Thackery [130] agreed with these findings and subsequently established that prions could not be expressed in insect genomes due to the fact that insects lack the PrP-encoding gene. However, it was agreed that insects could potentially act as mechanical vectors of prions, derived from at-risk substrates of ruminant origin. Concern was also expressed for humans and susceptible animals who may consume the contaminated products at a later stage within the food chain [38]. Finally, Pinotti [131] demonstrated that food waste could be used as an alternative source of nutrition for swine and cattle, which could potentially reduce the transfer of prions along the food chain.

#### 5.2.4. Parasites

Edible insects have been identified as a potential reservoir of human and animal parasites [132]. Protozoa are known to symbiotically reside in the gut of beetles, cockroaches, and termites where they facilitate the digestion of cellulose and lignin, after which there is the potential for these invertebrates to synthesize protein [2].

Protozoa parasites include Entamoeba histolytica, *Giardia lamblia*, *Toxoplasma* spp., and *Sarcocystis* spp all of which have been isolated from cockroaches. These parasites must be considered when consuming insects as a food source [15,132]. *Dicrocoelium dendriticum* is a zoonotic parasite which can be transmitted to humans via the consumption of edible insects such as ants or crickets, either raw or undercooked. Furthermore, *D. dendriticum*, also known as wire worm or horsehair worm, is the causative agent of a rare foodborne zoonosis of the human biliary tract. Dicrocoeliosis transmission to humans is via the oral route when ingesting the infested liver of ruminants (pseudodicrocoeliosis) or the ingestion of an ant or a cricket, that inadvertently acted as an intermediate host for the parasite during it’s lifecycle [133]. This parasite nematode, also known as *Gordius* spp., can grow to 1.5 m in length. Moreover, a new species of *Gordius chiashanus* spp. was identified in millipedes found in Taiwan [134]. Although they are not fatal to humans they can cause extreme discomfort, vomiting, and diarrhoea.

### 5.3. Allergens

An adverse immune response to food, which is caused by substances called allergens, a type of antigen, is referred to as a food allergy [135]. Symptoms can include itching, swelling, asthma, anaphylactic shock, or death [9]. Previously, Broekman [136] highlighted that a food allergy develops in two phases: Initially, in phase one, a human becomes sensitized to specific food allergens. During the second phase, it has been established that when sensitized patients were subsequently exposed to the respective allergen, an allergic reaction was elicited. Furthermore, Pali-Scholl et al. [48] found that an allergic reaction to food proteins was characterized by the presence of antigen-specific serum IgE antibodies, leading to the release of histamine among others.

The potential of allergies related to insects as a source of food may be based on the existence of pan-allergies to arthropods. An arthropod is an invertebrate animal that possesses an exoskeleton, a segmented body, and paired jointed appendages from the phylum Euarthropoda. According to Murefu [12], arthropods are related to crustaceans, and therefore arthropods, such as arachnids and myriapods, are recognized sources of induced allergic reactions in humans. Such reactions are often induced by the presence of tropomyosin, arginine kinase, glyceraldehyde 3-phosphate dehydrogenase, and haemocyanin.

Although insects can cause an allergic reaction via contact, inhalation, or oral ingestion, it was found that locusts, grasshoppers, and silkworm pupae are responsible for anaphylactic shock [136,137]. Similarly, research undertaken by Broekman [136,138] found that in humans, primary sensitization could manifest upon consumption of mealworms, although such a reaction did not imply that the consumer would automatically have an allergic reaction when presented with another species of an edible insect.

Cross-reactive allergies have been identified in crustaceans, cockroaches, and dust mites. These reactions were also observed in studies by de Gier and Jeong [139,140] where they identified some cross-reaction between house dust mite sensitivity and yellow mealworm proteins including insect tropomyosin and arginine kinase. These reactions were evident in individuals with previously established allergies to house dust mites and crustaceans.

De Gier and Verhoeckx [139] also noted that diagnostic allergy tests and vaccines were successful when based on recombinant allergens retrieved from cockroaches, silkworms, and Indian meal moths.

Research undertaken by Fukatomi [9] confirmed that insects were a major source of allergens to humans, despite their regional variation and domestication. It was found that inhalation of particles over a prolonged period, by insect farmers, was the main source of contamination, resulting in respiratory sensitization including asthma. A previous study by Pener [141] compartmentalized insect allergens into three categories: Firstly, the occupational allergy of personnel rearing and breeding insects. Secondly, allergic reactions to acridid aggregations in the field. And, thirdly, food allergy. It was also established that the peritrophic membrane of locusts, which is secreted in the gut and excreted as a wrapping around the faeces, was the main causative agent of occupational allergic reactions.

#### Gluten-Free Food Source

Mealworms are often reared on starchy substrates of wheat, spent grains, bread, and cookies [142]. Most of these substrates contain gluten. The mealworms are reared in nursery trays and are in continuous contact with the substrate which could possibly facilitate the transfer of gluten to the surface of the larvae present and subsequent migration of the gluten to the gut of the larvae. Therefore, this indicates that mealworms could possibly be a vehicle of gluten in the final processed product. However, if gluten-free substrate is employed in the rearing pen, this could mitigate the source of any potential cross contamination [10].

### 5.4. Physical Hazards

Caution is recommended regarding the consumption of specific parts of whole insects. The EFSA recommends that the wings and legs of whole crickets are discarded prior to consumption as they contain chitinous spines and cuticular hairs. The hazardous chitinous spines and cuticular hairs can potentially cause internal injury when ingested by the consumer. Furthermore, the sharp cricket wings can adhere to the teeth and damage the gums. The sharp wings can also become embedded in the throat if swallowed and induce choking. Therefore, the EFSA advises the labelling of whole insect products to adequately inform the consumer of the potential physical hazards associated with edible insects and also the appropriate “in home” practices to be used when handling, washing, cooking, and consuming edible insects [38].

## 6. Nutritional Composition of Edible Insects

Edible insects are a potential source of dietary protein, including but not limited to, essential amino acids, monounsaturated, and polyunsaturated fatty acids. They are also a rich source of micronutrients including calcium (Ca), copper (Cu), iron (Fe), magnesium (Mg), manganese (Mn), phosphorous (P), selenium (Se), zinc (Zn), and riboflavin, pantothenic acid, biotin, and folic acid [14,143,144]. However, the overall nutritional composition of edible insects is dependent on the metamorphic stage of the insect, their diet, responses to their immediate environment, and the final processing methods, including but not limited to, drying, boiling, frying, and roasting [145].

The bioavailability of macronutrients and micronutrients, as defined by Melse-Boonstra [146], refers to the ‘fraction of an ingested nutrient that becomes available for use and storage in the body’. However, the rearing and processing methods of edible insects can influence their potential macronutrient and micronutrient composition. Further research undertaken by Ojha [147] found that the bioaccessability of such nutrients released from a food matrix during digestion and absorption can vary. Therefore, from a nutritional and health perspective, the digestibility and metabolized components of edible insects are the key aspects to be considered [148,149,150]. Further research on the bioavailability of macronutrients and micronutrients would be beneficial as there is a paucity of knowledge within this area.

Furthermore, insect farming and production has the potential to positively contribute to “Transforming our world: the 2030 agenda for sustainable development”, as defined by the UN [14,29]. Insects are regarded as poikilotherm animals which are cold-blooded and therefore have a high feed conversion rate which enhances their efficiency in biotransformation processing of organic matter into insect biomass [133,134]. Almost 80% of the mass of most insects can be consumed and digested, whereas only 55% of chicken and pork, and 40% of cattle is consumed [31,135].

### 6.1. Macronutrients

A recent study conducted by Pal [151] found that the macronutrient composition of beef and crickets were comparable as crickets contained 205 g of protein and 68 g of fat per kg, while ground beef contained approximately 256 g of protein and 187 g of fat per kg. A similar study previously undertaken by Rumpold and Schluter [152] identified the macronutrient composition of potential edible insects. Meanwhile, Ordonez-Araque [34] recently reviewed the kcal composition of the same selection of edible insects, the results of which are tabulated in Table 7.

#### 6.1.1. Protein

Protein is globally recognized as a component of a healthy diet for humans [30]. The current increased demand for protein is in part due to socioeconomic changes, including but not limited to rising incomes, increased urbanization, and aging populations [151]. The protein content of invertebrates is derived from a range of amino acids found within insects, while the quality of the protein is determined by the presence of essential or non-essential amino acids, although the digestibility of the proteins must also be considered [145].

The Kjeldahl method is widely used to quantify the crude protein present in the insect, where content can range from 8% to 70% of dry mass [153,154]. The Dumas technique is also used to determine protein content [155]. Both techniques, which focus on nitrogen content, are recognized as standard methods of analysis. However, not all nitrogen contained in insects originates from proteins [35]. Therefore, it is possible that digestible protein content calculated in insects using the aforementioned methods may be overestimated. Previous research by Bosch [156] highlighted that some differences in protein digestibility from different insects resulted from different cuticular protein sclerotization. Thus, protein content calculated using the Kjeldahl analysis and conversion factors developed for other foods would be expected to overestimate the protein content of the whole insect, as it does not distinguish between easily digested proteins, inaccessible proteins, chitin, and other Nitrogen-rich molecules [153].

#### 6.1.2. Fat

The fat content of invertebrates is high in monounsaturated and/or polyunsaturated fatty acids. Essential fatty acids, including the omega-3 fatty acids of α-linolenic acid and the omega-6 fatty acids of linoleic acid, are also present in insects. However, fat content can differ between species and even within a single species due to environmental factors, contaminants and, in particular, the individuals’ uptake of heavy metals [1,145]. Similarly, Bawa [148], for example, found that the fat content of *A. domesticus* was subsequently altered depending on the diet they consumed.

#### 6.1.3. Fibre

Over consumption of locusts and grasshoppers without removing the legs can cause severe constipation in humans due to the large spines present on the tibia of the insects which can become embedded in the human the gut [1,141]. Furthermore, surgery is often required to remove the undigested spines [157]. Similarly, when patients in eastern Java, Indonesia, overconsumed roasted scarab beetles (*Lepidiota* spp.) surgery was also necessary to relieve total constipation caused by indigestible chitinous accumulation within the human gut. [1,158]. Therefore, when insects are consumed within the recommended limit of less than 30% of plate portion content they can potentially contribute positively to human consumption of fibre without any resulting constipation.

### 6.2. Antinutrients

Fibrous chitin is a structural nitrogen-based carbohydrate which is present in the exoskeleton of insects. Chitin may contain anti-nutrient properties related to negative effects associated with protein digestibility [15]. However, further research by Rumpold and Schluter [31] found that although chitin is considered indigestible by humans [159], chitinolytic enzymes produced by bacteria located in the human gastrointestinal tracts indicated that chitin and chitosan can be digested by humans. Furthermore, nutrient intake can also be reduced by the consumption of antinutrients such as tannin, oxalate, hydrocyanide, and phytate, all of which can be found in varying degrees in edible insects [71].

### 6.3. Micronutrients

Micronutrient levels vary greatly within insect species depending on their age and the development stage of their life cycle, all of which can also be influenced by their immediate environment [31,61,160]. 

#### Vitamins and Minerals

**Figure 2:**
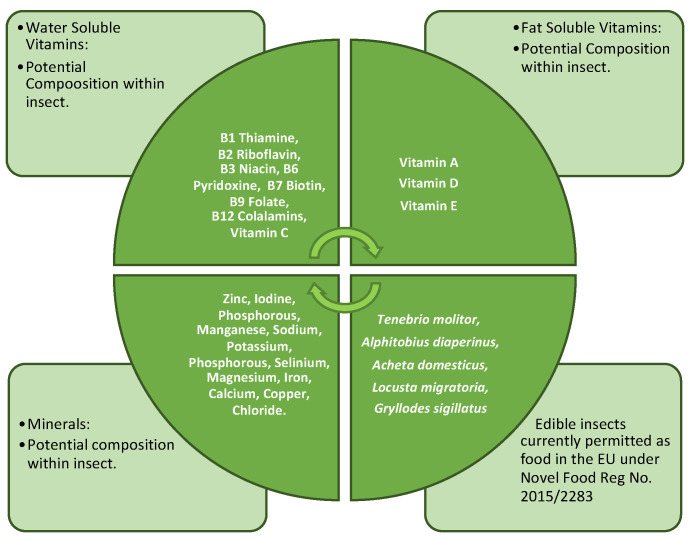
Micronutrients potentially available in edible insects.

Environmental factors, contaminants, and metals acquired during a life cycle and processing exposure can influence the final mineral and vitamin content of the insect. Moreover, research conducted by Mattia [37] found that crickets and grasshoppers displayed antioxidant values superior, by up to three-fold, to those of orange juice and olive oil. Furthermore, Vitamin B12 occurs only in food of animal origin and can be found in *T. molitor* at 0.47 μg per 100 g and in *A. domesticus* at 5.4 μg per 100 g in adults and 8.7 μg per 100 g in nymphs [2,159,161]. A potential combination of micronutrients found in edible insects can be viewed in Figure 2.

## 7. Legislative Requirements

There are several mandatory legislative requirements that directly impact the rearing, harvesting, and processing of insects as food and feed. Foodstuffs produced with new technologies, derived from new sources, new substances, and traditional foods consumed in non-EU countries, not consumed to a significant degree within the EU before 15 May 1997, are defined as “novel foods” under Regulation (EU) 2015/2283 [32]. However, current concerns regarding a sustainable future foodscape, climate change, and environmental erosion are further addressed in the European Farm-to-Fork strategy as defined by the European Commission [162].

The current Novel Food Regulation (EU) 2015/2283 was implemented from 1 January 2018 [32]. Under current legislation, food business operators can place authorized novel foods on the European Union Market for sale if specifications are also adhered to, as previously outlined in Regulation (EC) 1169/2011, and as advised by the European Food Safety Authority [163,164]. The EFSA have been involved in the risk assessment of foods since 2003 [38].

EU legislation follows the precautionary principle through Regulation (EC) No. 178/2002 [165], which states that, if potential risks from consumption of new foods are identified, a premarket risk assessment has to be performed. However, in the context of Regulation (EC) 853/2004, concerning animal foodstuffs, frogs legs, and snails, which are mentioned among the “unconventional” foods of animal origin; interestingly, insects were not included within this category [101].

The main adjustments from the original novel food Regulation (EC) No. 258/97 [166] to the current novel food Regulation (EU) No. 2015/2283 [32], as observed by de Boer and Bast [80], were firstly concerned with updating the definition of novel foods, where currently Article 3 of Regulation 2283/2015 effectively defines a novel food based on two elements, firstly whether it has not been consumed to a significant degree within the EU before 15 May 1997 and, secondly, if it falls within one of the defined categories of novel foods [32]. Secondly, a centralized authorization procedure was introduced and is currently available through the EFSA, instead of through the member state selling the product, as per original procedure requirements [166,167], and the establishment of a Union list of authorized novel foods with a generic authorization decision was agreed [38]. Finally, the immediate involvement of the EFSA in the risk assessment process was also vetoed by member states.

Originally Regulation (EC) 258/97 was developed to ensure that new food products entering the market that originated from outside the EU or that were processed using either new technologies or scientific findings remained safe for human consumption.

Regulation (EC) 258/97 also defined a clear regulatory framework for novel foodstuffs to stimulate trade and innovation within the internal European market [166,168]. Therefore, insects and insect-based food and feedstuffs must adhere to legislation as per all permitted food and feedstuffs within the European Union [169,170].

## 8. Sustainable Insect Farming

There are three types of insect farming: wild harvesting, semi domestication, and closed farming. Closed farming in Europe is a highly regulated and upscaled vertical farming production process, designed specifically for the rearing and harvesting of large numbers of insects as a sustainable food source suitable for human consumption. Currently, industrial insect processes are undergoing automation in Europe, with a view to further upscaling sustainable production and increasing profits within the food and feed sector [171]. Furthermore, there is potential for this trend to positively contribute to future food security and the circular economy [2]. However, not all insect species are suitable for rearing under intensive automated vertical farming methods [172].

The collection and harvesting of wild insects often leads to damage to their habitat and the risk of collecting individuals containing dangerous levels of heavy metals and pesticides [1,11]. Moreover, insects caught in the wild are often eaten raw or minimally cooked, thus potentially creating a food safety risk to the consumer. Insect farmers and their families often rear domesticated insects and larvae on a small scale to increase or supplement the family income.

However, small scale rearing is currently being replaced by upscaled production in Europe. In 2019, European insect food business operators (iFBO’s) produced approximately 500 tonnes of insect-based products for sale on the European market and it is expected that 260,000 tonnes of insect-based products will be required to meet a growing demand in Europe by 2030 [173]. Currently, yellow mealworms (*T. molitor*), lesser mealworm (*A. diaperinus* larvae), migratory locusts (*L. migratoria*), and house crickets (*A. domesticus*) are approved as food-grade in compliance with EU novel food legislation and are successfully farmed in Europe without any recorded adverse impact regarding local ecosystems. Meanwhile, tropical house crickets (*G. sigillatus*) are pending approval. However, caution must be advised regarding *A. diapernius* larvae as this species has the potential to become a pest upon escape from the farm due to its’ ability to burrow and survive in adverse conditions.

### 8.1. Vertical Farming

Vertical farming under controlled conditions can contribute positively to the food safety aspect of the product, its cost-efficiency, energy-efficiency, food security, and sustainability [2,171]. Previously, a study by van Broekhoven [174] found that under controlled conditions during production, alterations in the diet of the larvae of yellow mealworms (*T. molitor*), could alter the fatty acid profile of the larvae without altering the protein profile of the larvae. Moreover, Pinotti and Ottobini [131] agreed that manipulation of the insect substrate could also influence the growth and life cycle of insects, in particular black soldier fly (*Hermetia illucens*). Similarly, parasites and pathogens can be controlled and mitigated by adhering to current mandatory legislation, including traceability. Therefore, uncontaminated stock reared under strict HACCP conditions within the “vertical farming method” could possibly offer a safe alternative and sustainable source of proteins and fats to potential consumers. Furthermore, insects as a food source can potentially contribute positively to the circular economy while simultaneously reducing the carbon footprint of protein production by feeding on waste streams [2]. However, not all insects are suitable for rearing under the vertical farming method.

### 8.2. Upscaled Production and Slaughter

Consideration must be given to the mass numbers of insects that could potentially be reared within an upscaled, automated, and controlled environment [175]. Such insect numbers, unless effectively managed, could induce stress within the insect colony and ultimately initiate infection related diseases via cannibalism and excretion of pathogens and previously dormant pathogenic spores, residing within the insect gut. Therefore, a starvation period is recommended prior to slaughter to reduce the spread of infection. Previously, Castex [176] identified the relationship between arthropods and crustaceans including but not limited to shrimp. This relationship was further explored to establish the potential use of probiotics in shrimp aquaculture as a means of enhancing the arthropods’ immune response against *Vibrio* spp. This method, according to Maciel-Vergara [126], merits investigation as, due to the relationship between insects and shrimp, there is the potential that insects could possibly develop immune responses to pathogens if reared on shrimp-derived probiotics, which, in turn, could possibly reduce pathogen count in arthropods post-slaughter. Furthermore, Oonincx and Finke [145] agreed that it is possible to manipulate the nutritional composition of insects, while Fernandez-Cassi [65] found that starvation of the mini-livestock prior to slaughter reduced the microbial load of some insects. Furthermore, Imathiu and Ojha [7,177], agreed that the regulated processing of insects is crucial for the production of a safe and palatable insect-based foodstuff.

### 8.3. Food Security and Sustainability and the Circular Economy

Insects currently offer a low carbon footprint during the rearing and harvesting cycle due to low greenhouse gas emissions, low ammonia emissions, and low water consumption [14,178]. Several species are effectively reared on organic waste and are suitable candidates for sustainable and intensive rearing methods [3]. Therefore, insects have the potential to create future food security within a sustainable circular economy [2].

### 8.4. Insect Welfare

**Figure 3:**
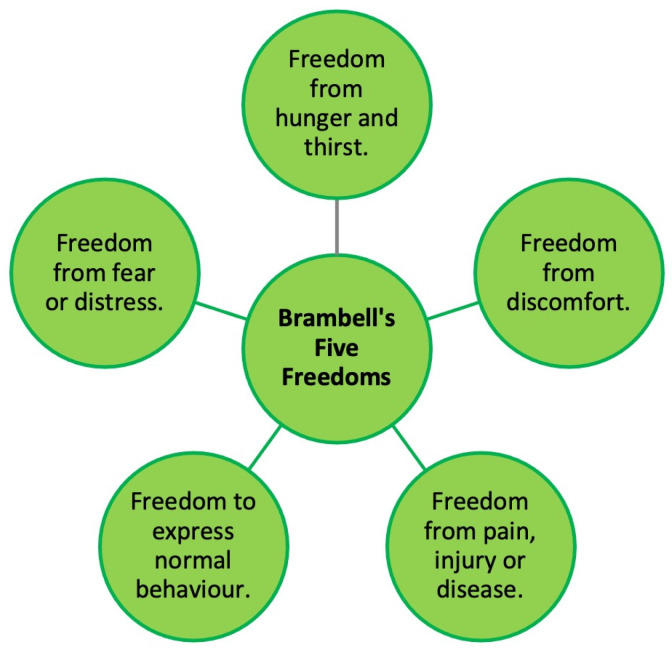
Brambell’s five freedoms.

Recently, Arnold van Huis [179] reviewed the importance of insect welfare and highlighted that it must be addressed and implemented at all times, while Maciel-Vergara [64] focused on colony health where insect welfare standards and analytical methods for detection are used within the sector to minimize production losses via pathogens within farmed insect populations. Currently, the most reliable method of quantitative research would be the real-time PCR method [132]. Furthermore, overcrowding in pens/trays must be monitored to reduce cannibalism, pathogen excretion, and subsequent contamination of the mini-livestock [179]. Therefore, the five freedoms (Figure 3) as originally defined in 1965 by Brambell [180], and further supported by Poletto and van Huis [179,181] are recommended during all stages of the insects’ lifecycle, to facilitate and maintain the humane rearing of this farmed mini-livestock.

## 9. Future Perspective and Conclusions

Insects could potentially become a palatable, safe, and sustainable food source. They contain sufficient levels of safe macronutrients and micronutrients suitable for human consumption and offer a low carbon footprint, increased economic value, and have the potential to positively contribute to a sustainable circular economy. However, upscaling insect production would need to be further explored and a market for such produce would also need to be established. Consumer perceptions in Europe are currently adversely influenced by cultural norms and neophobia while potential issues regarding insects and insect-based foodstuffs are similar to those of currently established marketable foodstuffs, including both fresh and processed products. Insects and insect-based foodstuffs need to be introduced to consumers, as a palatable, safe, and sustainable food source for the future, as the benefits of edible insects cannot be fully realized until citizens choose to engage with entomophagy. There is a paucity of knowledge regarding the benefits of consuming insects and insect-based foodstuffs. Consumer education and familiarity with entomophagy will be required to enhance the journey of the mini-livestock from cradle to fork in a palatable and sustainable manner to further develop a nutritious and palatable end-product. Furthermore, an increased research effort on the cognitive and emotional capacity of invertebrate species is also required to fully understand insect welfare requirements for a healthy life.

## Figures and Tables

**Table 1:** Selection of edible insects defined using binomial nomenclature and common names.

Order	Binomial Nomenclature	Common Name	References
Coleoptera	*Alphitobius diaperinus*	Lesser mealworm; Litter beetle; Buffalo mealworm.	[25,26,27,28,29]
Coleoptera	*Rhynchophorus ferrugineus*	Palm weevil.	[1,26,30,31,32,33,34,35,36]
Coleoptera	*Alphitobius laevigatua*	Black fungus beetle.	[34]
Coleoptera	*Tenebrio molitor*	Yellow mealworm.	[26,37,38,39,40,41,42]
Coleoptera	*Zophobas atratus*	Giant mealworm.	[26,30,42,43,44]
Diptera	*Chrysomya chloropyga*	Blowfly.	[26,30]
Diptera	*Hermetia illucens*	Black soldier fly.	[26,42,45,46,47]
Diptera	*Musca domestica*	Housefly.	[26,42]
Hymenoptera	*Apis mellifera*	European honeybee.	[26,42,48,49,50]
Hymenoptera	*Atta laevigata*	Leafcutter ant.	[3,26,33,51]
Lepidoptera	*Achroia grisella*	Lesser wax moth.	[10,26,30,51]
Lepidoptera	*Bombyx mori*	Silkworm/domestic silk moth.	[26,42,47]
Lepidoptera	*Galleria mellonella*	Greater wax moth.	[26,42,52]
Lepidoptera	*Gonimbrasia belina*	Mopane worm/caterpillar.	[26,30,53,54,55]
Orthoptera	*Acheta domesticus*	House cricket.	[26,42,56,57,58,59,60]
Orthoptera	*Gryllodes sigillatus*	Tropical house cricket; Indian house cricket; Banded house cricket.	[26,42,61,62]
Orthoptera	*Gryllus assimilis*	Jamaican field cricket.	[63,64]
Orthoptera	*Gryllus bimaculatus*	Two-spotted cricket.	[26,65,66,67]
Orthoptera	*Gryllus campestris*	European field cricket.	[26,57,68]
Orthoptera	*Locusta migratoria* *migratorioides*	Migratory locust; European migratory locust; African migratory locust.	[26,32,42,51,69,70]
Orthoptera	*Mecopoda elongate*	Bush cricket.	[26,34]
Orthoptera	*Oxya* spp. *Melanophus* spp. *Hieroglyphus* spp. *Acrida* spp.	Grasshopper.	[34]
Orthoptera	*Patanga succincta*	Bombay locust.	[34]
Orthoptera	*Schistocerca americana*	American grasshopper.	[34]
Orthoptera	*Schistocerca gregaria*	Desert locust.	[26,42]
Orthoptera	*Teleogryllus mitratus*	Common cricket.	[34]

**Table 2:** Edible insects approved for human consumption within the European Union.

Order	Family	Genus	Species	Common Name	References
Coleoptera	Tenebrionidae	Tenebrio	*Tenebrio molitor*	Yellow Mealworm.	[71,72]
Coleoptera	Tenebrionidae	Alphitobius	*Alphitobius* *diaperinus* *larvae*	Lesser mealworm; Litter beetle; Buffalo worm.	[72,73]
Orthoptera	Gryllidae	Acheta	*Acheta* *Domesticus*	House cricket.	[72,73,74]
Orthoptera	Gryllidae	Gryllodes	*Gryllodes* *Sigillatus*	Tropical house cricket; Indian house cricket; Banded cricket.	[72,73]
Orthoptera	Acrididae	Locusta	*Locusta* *Migratoria*	African migratory locust; European migratory locust.	[71,72]

**Table 3:** Insect-based food products previously for sale at retail outlets in Europe.

Insect	Product	Retailer
Mealworms	Buggy balls	Dutch retailer Jumbo, the Netherlands.
Buffalo Worms	Buggy citizens	Jumbo, the Netherlands.
Waxworm Larvae	Buggy crisps	Jumbo, the Netherlands.
Dutch Bred Buffalo Worms	Insecta Range: Burgers and schnitzels and nuggets	Food producer Damhert Nutrition, Belgium.
Crickets	SENS bar	Czech-based food start-up in 2017.
Crickets	Energy bars; Bag of crunchy roasted crickets in different flavours.	‘EAT GRUB’ is a food brand available online. T: 0203 633 5771 E: info@eatgrub.co.uk https://www.eatgrub.co.uk/ (accessed on 10 January 2024).
Crickets or Mealworms	Bolognese sauce.	One Hop Kitchen: https://www.instagram.com/onehopkitchen/ (accessed on 10 January 2024).

**Table 4:** SENS edible insect-based food products currently available online.

Insect	Product	Flavour	Online Retailer
Crickets.	Roasted Edible Crickets; Snack Gift Box.	Spicy and Sweet; Chocolate; Extra hot.	SENS https://www.eatsens.com (accessed on 10 January 2024).
Mealworms.	Crunchy Edible Worms; Gift Set.	Garlic and herbs; Onion and parsley; Smoked paprika; Chilli and lime.	SENS https://www.eatsens.com (accessed on 10 January 2024).
Crickets.	Edible Crickets in a Tube.	BBQ; Tomato and oregano; Salty caramel; Dark chocolate; Milk chocolate; White chocolate; Chocolate and cinnamon; Chilli and lime; Wasabi; Chipotle and carolina; Reaper.	SENS https://www.eatsens.com (accessed on 10 January 2024).
Crickets	Edible Crickets in XXL Bag.	Chilli and lime; BBQ paprika; Tomato and oregano; Salty caramel	SENS https://www.eatsens.com (accessed on 10 January 2024).
Crickets	Pea Cricket Protein Chips	Poppy seed and sea salt; Hot paprika; Garlic and herbs.	SENS https://www.eatsens.com (accessed on 10 January 2024).
Crickets	Sustainable Sports Nutrition; Protein Blend.	Chocolate; Strawberry.	SENS https://www.eatsens.com (accessed on 10 January 2024).
Crickets	Cricket Protein Bar.	Dark chocolate.	SENS https://www.eatsens.com (accessed on 10 January 2024).
Crickets	Serious Cricket Protein Bar.	Bitter cocoa and sesame.	SENS https://www.eatsens.com (accessed on 10 January 2024).
Crickets	Pleasure Cricket Protein Bar.	Pineapple and coconut; Dark chocolate and orange.	SENS https://www.eatsens.com (accessed on 10 January 2024).
Crickets	Oat Cricket Protein Breakfast.	Apple and cinnamon; Hazelnut.	SENS https://www.eatsens.com (accessed on 10 January 2024).
Crickets	Ready-to-Eat Outdoor Meal; Cricket Protein with Penne.	Vegetables; Chicken.	SENS https://www.eatsens.com (accessed on 10 January 2024).
Crickets	Cooking and Baking; Cricket Protein Powder; Cricket Protein Pasta.	Unflavoured.	SENS https://www.eatsens.com (accessed on 10 January 2024).

**Table 5:** Eat Grub edible insect-based food products currently available online.

Insect	Product	Flavours	Retailer
Crickets, buffalo worms, mealworms, grasshoppers.	Ready-to-Eat Bundle; All in(sect) pack. Single species per pouch.	Smoky BBQ; Peri-peri; Chilli and lime; Unflavoured.	Eat Grub https://www.eatgrub.co.uk (accessed on 10 January 2024).
Crickets.	Ready-to-eat bundle; Crunchy Roasted Crickets; Each pouch contains a different flavoured cricket; Classic Combo; Smokin’ hot.	Salted toffee; Salt and vinegar; Peri-peri; Smoky BBQ; Sweet chilli and lime.	Eat Grub https://www.eatgrub.co.uk (accessed on 10 January 2024).
Crickets, grasshoppers, mealworms, buffalo worms.	Cooking Pack bundle; Edible insects Starter pack; Foodie pack; Freeze-dried insects.	Unflavoured.	Eat Grub https://www.eatgrub.co.uk (accessed on 10 January 2024).
Crickets, mealworms, buffalo Worms.	Ingredients: Cricket Protein/Flour; Edible Crickets for roasting; Edible mealworms for roasting; Buffalo Worms for frying.	Unflavoured.	Eat Grub https://www.eatgrub.co.uk (accessed on 10 January 2024).
Crickets.	Snacks Ready-to-Eat; Crunchy Roasted Crickets; The big mix; Individual tubes; Individual XXL bags.	Peri-peri; Classic combo; Smokin’ hot. Salt and vinegar; Salted toffee; Smoky BBQ; Sweet chilli and lime.	Eat Grub https://www.eatgrub.co.uk (accessed on 10 January 2024).

**Table 6:** One Hop Kitchen edible insect-based food products currently available online.

Insect	Product	Flavours	Retailer
Crickets.	Cricket Bolognese Sauce.	Combination of meat, herbs, and sweet tomatoes.	One Hop Kitchen https://www.instagram.com/onehopkitchen/ (accessed on 10 January 2024)
Mealworms.	Mealworm Bolognese Sauce.	Combination of meat, herbs, and sweet tomatoes.	One Hop Kitchen https://www.instagram.com/onehopkitchen/ (accessed on 10 January 2024).

**Table 7:** Macronutrient composition of edible insects.

Order	Family	Insect 100 g	Protein	Fat	Fibre	kcal	Refs
Orthoptera	Gryllidae	*Acheta domesticus*	64.10%	24%	6.20%	--	[20,26]
Orthoptera	Acrididae	*Acrida exaltata*	64.46%	7.07%	7.73%	336.93	[20,26]
Orthoptera	Acrididae	*Arphia fallax*	71.30%	6.52%	11.58%	367.04	[20,26]
Orthoptera	Acrididae	*Melanoplus femurrubrum*	77%	4.20%	12.10%	370.00	[20,26]
Coleoptera	Tenebrionidae	*Tenebrio molitor* (adult)	60.20%	20.80%	16.30%	460.60	[20,26]
Coleoptera	Tenebrionidae	*Tenebrio molitor* (pupal)	53.10%	36.70%	5.10%	552.90	[20,26]
Hempitera	Coreidae	*Pachylis gigas* (adult)	65%	19%	10%	451.00	[20,26]
Hempitera	Coreidae	*Pachylis gigas* (nymph)	63%	26%	5%	496.00	[20,26]
Lepidoptera	Eribidae	*Latebraria amphipyrioides*	57%	7%	29%	349.00	[20,26]
Lepidoptera	Hepialidae	*Phassus triangularis*	15%	77%	4%	761.00	[20,26]
Lepidoptera	Notodontidae	*Anaphe venata* (larvae)	60.03%	23.22%	2.30%	453.70	[20,26]
